# Comprehensive Analysis and Characterization of Linear Antigenic Domains on HN Protein from Genotype VII Newcastle Disease Virus Using Yeast Surface Display System

**DOI:** 10.1371/journal.pone.0131723

**Published:** 2015-06-29

**Authors:** Tao Li, Gaoling Wang, Bingtian Shi, Peixin Liu, Wei Si, Bin Wang, Li Jiang, Lunjiang Zhou, Jinsheng Xiu, Henggui Liu

**Affiliations:** 1 State Key Laboratory of Veterinary Biotechnology, Harbin Veterinary Research Institute, the Chinese Academy of Agricultural Sciences, Harbin, China; 2 College of Animal Sciences, Fujian Agriculture and Forestry University, Fuzhou, China; 3 Institute of animal husbandry and veterinary medicine, Fujian academy of agriculture sciences, Fuzhou, China; Sun Yat-sen University, CHINA

## Abstract

Circulation of genotype VII Newcastle disease virus (NDV) has posed a great threat for the poultry industry worldwide. Antibodies against Hemagglutinin-neuraminidase (HN), a membrane protein of NDV with critical roles in NDV infection, have been reported to provide chickens protection from NDV infection. In this study, we comprehensively analyzed the in vivo antibody responses against the linear antigenic domains of the HN protein from genotype VII NDV using a yeast surface display system. The results revealed four distinct regions of HN, P1 (1-52aa), P2 (53-192aa), P3 (193-302aa) and P4 (303-571aa), respectively, according to their antigenic potency. Analysis by FACS and ELISA assay indicated P2 to be the dominant linear antigenic domain, with the immunogenic potency to protect the majority of chickens from NDV challenge. In contrast, the P1, P3 and P4 domains showed weak antigenicity in vivo and could not protect chickens from NDV challenge. These results provide important insight into the characteristic of humoral immune responses elicited by HN of NDV in vivo.

## Introduction

Newcastle disease (ND) is a highly contagious and widespread disease. It has been a great threat to the poultry industry, resulting in huge yearly economic losses since its emergence. ND is caused by infection with the Newcastle disease virus (NDV), which belongs to the genus *Avulavirus* of the family *Paramyxoviridae* (http://ictvonline.org). NDV has a single-stranded, negative-sense, nonsegmented RNA genome with 15186, 15192 or 15198 nucleotides in length [1–3]. Its genome contains six genes which encode for the nucleoprotein (NP), phosphoprotein (P), matrix (M), fusion (F), hemagglutinin-neuraminidase (HN) and the RNA-dependent RNA polymerase (L) in the 3’ to 5’ orientation [4]. The virulence of different NDV isolates varies remarkably. Based on their pathogenicity, they are grouped into three classes: the lentogenic viruses which are the least virulent and cause asymptomatic infection; the mesogenic viruses which are moderately virulent and typically present with respiratory or neurological signs and the velogenic viruses which are the most virulent and are often fatal due to extensive necrosis and hemorrhaging [5]. Historically, NDV isolates have been classified into nine genotypes, genotype I to IX, based on the phylogenetic analysis of the partial or complete nucleotide sequence of the F gene [1, 6–13]. Clinical investigations have shown NDV to be evolving. In the last century, genotypes V and VI have been the dominant strains circulating within the poultry industry. In more recent decades, genotype VII and VIII have emerged as the dominant cause of deathly infection in all kinds of poultry [13, 14]. In particular, genotype VII has become the predominant circulating virus and has recently been isolated in broiler, layer and breeder farms in Jordan, duck flocks in China, live-bird markets in Nigeria, pheasant farms in Spain, and poultry farms in Malaysia [15–18].

HN is the membrane protein of NDV and plays pivotal roles during host viral infection, including receptor binding, and neuraminidase and fusion promotion activities [19–23]. Because of its critical roles in viral infection, antibodies against HN are crucial for the hosts’ ability to protect itself against NDV infection. Thus, HN has been a target of vaccine design [24]. Although we know that humoral responses elicited by HN are important for host protection from NDV infection, it remains unclear which domains on the HN protein can elicit these responses and what roles the individual antigenic domains play in host protection. Exploring these issues will provide important information for the future development of a new generation of vaccines against the circulating genotype VII NDV. As there is yet no established research platform to address these questions, we develop a yeast surface display system for our in vivo analysis of antibody responses against HN. Yeast surface display is a powerful means for protein engineering [25]. It acts by protein fusion to the adhesion subunit of the yeast agglutinin protein Aga2p, which attaches to the yeast cell wall through disulfide bonds to Aga1p [25]. So far, yeast surface display has been successfully used for antibody engineering [26, 27], antibody screening against a variety of antigens [28–31], and T cell receptor engineering [32]. Recently, it was also successfully used for the comprehensive antigenic analysis of viral proteins [33, 34]. The yeast surface display system is able to provide both qualitative and quantitative measurements of polyclonal responses in vivo in the field of antigenic analysis. Thus, the data obtained through the yeast surface display system will be able to identify specific antigenic domains of pathogens preferentially recognized in vivo and provide insights into the antigenic variation of a giving virus protein [33, 35]. Here, we defined the linear antigenic domains on the HN protein from genotype VII NDV using yeast surface display and further analyzed their immunogenicity and protective functions against NDV infection in vivo.

## Materials and Methods

### Antibodies, plasmid and virus

FITC-labeled goat anti-Chicken IgY and chicken anti-C-Myc polyclonal antibody (mAb) were purchased from Life Technologies. Plasmid pCTCON-2 and yeast strain *S*. *cerevisiae* EBY-100 for yeast surface display were kindly provided by Dr. Linqi Zhang (Tsinghua University). The NDV genotype VII strain JS2012 was isolated by our laboratory and propagated and titrated in 9-day-old embryonated specific-pathogen-free (SPF) eggs as previously described [36].

### Preparation of hyperimmune sera against genotype VII NDV in chickens

To prepare the chicken immunosera against genotype VII NDV, strain JS2012 in allantoic fluid was purified by centrifugation at 28000g for 12h with a 20% sucrose cushion. The purified NDV were emulsified with an equal volume of complete or incomplete Freund’s adjuvant (Sigma-Aldrich) after inactivation with a 0.02% final concentration of β-propiolactone, and inoculated into one month old SPF chickens through muscle injection. The chickens were boosted with the live virus after the immunization twice with two week intervals. The immunosera were prepared on day 30 after the last immunization after being drawn from the heart and titrated using HI assay. All experiments involving experimental chickens were approved by the Animal Ethics Committee of Harbin Veterinary Research Institute (HVRI), and conducted according to HVRI guidelines.

### Construction of the combinatorial HN antigen yeast library from genotype VII NDV

To construct the HN antigen yeast library, plasmid pCTCON-2 was modified with additional T-overhang (pCTCON-T) as described previously [33]. Construction of the library was performed as previously described with only slight modification [33, 34]. Briefly, the full-length HN gene from genotype VII NDV strain JS2012 was amplified by polymerase chain reaction (PCR) using the primers HN-F (5’-ATGGACCGCGCGGTTAACAGAGT-3’) and HN-R (5’-AACTCTATCATCCTTGAGGATCTC-3’). After purification with a DNA gel extraction kit (Thermo Fisher Scientific), the PCR-amplified products were randomly digested with a final concentration of 0.1 unit DNase I to obtain fragments between 50–300 bp in length. The obtained randomly-digested DNA fragments were A-tailed with Taq polymerase and ligated into the pCTCON-T vector. The ligation products were transformed into competent EBY-100 yeast cells by electroporation. The transformed cells were titrated onto SDCAA Amp plates at 30°C to determine the yeast library size. The recombinant plasmids were extracted from a part of yeast clones randomly selected on the SDCAA Amp plates and sequenced to determine the quality of the combinatorial HN antigen yeast library.

### Immunofluorescence staining and sorting of combinatorial HN antigen yeast library

The combinatorial HN antigen yeast library from genotype VII NDV was induced with 2% galactose as described previously [26]. The random peptides of HN expressed on the yeast surface were stained with the prepared chicken immunosera against genotype VII NDV as described previously [33, 34]. In brief, 10^6^-10^7^ induced yeast cells were pelleted by centrifugation at 3000 g, resuspended with 100 μl of chicken immunosera against NDV diluted 1:200 with PBS and incubated at 4°C for 30 min. The stained cells were washed three times with PBS and then stained with FITC-labeled anti-chicken IgY secondary antibodies at 4°C for another 30 min. Finally, the cells were washed three times and resuspended in PBS. To adjust the parameter of the cytometer for FACS analysis and sorting, the yeast cells expressing HN antigenic peptides were stained with FITC-labeled anti-chicken IgY secondary antibodies as described above to serve as the control. The yeast cells showing increased florescence intensity compared to the control in the FACSAria-II cytomyter (BD bioscience) were sorted as the positive cells. A total of 1×10^4^ cells were sorted from the library.

### Algorithm for sequence scanning and clustering

The positive yeast cells were submitted to plasmid extraction and sequencing. The obtained DNA sequences of HN antigens were translated into peptide sequences. The potential antigenic domains on the HN protein were analyzed using a novel computer algorithm developed by Dr. Linqi Zhang’s group as described previously [33]. In brief, a sliding window of 10 amino acids was used to scan across the entire alignment from the N to C terminus one residue at a time. When a window found fragment sequences containing at least five amino acid residues or was identical to ≥ 50% of the full length sequence, the position of the given window was scored based on the number of fragment sequences identified. The window containing the highest number of fragment sequences was classified as the first antigenic domain, which was then plotted based on the frequency of amino acid residues along their corresponding positions in the full-length HN sequence. The scanning process continued again from the beginning until the entire fragment sequences had been assigned to appropriate antigenic domains [33].

### Expression of antigenic domains of HN protein on the yeast surface and in *E*. *Coli*.

To express the defined antigenic domains on the yeast surface, individual fragments were amplified from the HN gene and inserted between the NheI and BamHI sites on the pCTCON-2 vector. The recombinant plasmids were then transformed into competent EBY-100 cells using electroporation. The expression of antigenic domains was induced with galactose as described above and analyzed with FACS after staining with antibodies against C-Myc tag. After confirming the expression of each antigenic domain on the yeast surface, the staining of antigenic domains by chicken immunosera against NDV were performed and analyzed with FACS as described above. To express the antigenic domains in E. *Coli*., the antigenic domain fragments were inserted between the EcoRI and XhoI sites on pGEX-6P-1. Protein expression was induced with 1 mM IPTG in *E*. *Coli*. BL21 (ED3+) and confirmed through SDS-PAGE assay. To purify the target protein, *E*. *Coli*. cells were first put through ultrasonication and the total cell lysate was run on an the SDS-PAGE gel. The band containing the target protein was precisely excised from the gel and crushed in PBS for protein release. The purity of the purified protein was confirmed by staining with Coomassie Brilliant Blue R-250 (Sigma-aldrich) in the SDS-PAGE gel.

### ELISA assay

To evaluate the antibodies against each antigenic domain, ELISA assay was performed as described previously [37]. In brief, 1 μg of antigenic proteins and GST expressed in *E*. *Coli*. were coated on ELISA plates (Thermo Fisher Scientific) in 100 μl 0.05 M carbonated bicarbonate (pH 9.6) by overnight incubation at 4°C. After washing four times with PBST (PBS containing 0.05% Tween-20), the plate was blocked with 5% skim milk at 37°C for 1 h and washed with PBST as above. A total of 100 μl chicken immunosera against genotype NDV diluted 1:200 with PBS was added to the indicated wells and incubated at 37°C for 1 h. The plate was washed again. Horseradish peroxidase (HRP)-conjugated goat anti-chicken IgY (Sigma-Aldrich) was added to each well and incubated at 37°C for 1 h. After washing again as above, 100 μl TMB substrate was added to each well. The reaction was stopped using 50 μl 0.5 N H_2_SO_4_ after incubation at room temperature for 30 min. The OD_450_ value of each well was immediately read with a microplate photometer (ELx800, BioTek).

### Immunization and challenge of chickens

The antigenic regions and HN gene deleted the signal peptide were cloned in one-frame into eukaryotic expression vector pVAX-S-Flag which contains the signal sequence from human PD-1 at the 5’-terminal and Flag tag at the 3’-terminal of the multiclonal site. To confirm the expression of the individual proteins in mammalian cells, the recombinant plasmids were transfected into 293T cells. The antigenic proteins and HN in the supernatant were harvested through precipitation with 4 times the volume of acetone 48 h post transfection, and detected with anti-Flag mAb using dot-blot assay. To immunize chickens with the recombinant plasmids, 15 day old of 50 SPF White Leghorn chickens were randomly divided into six equal groups and fed in BSL2 experimental animal facilities in the animal centre of HVRI. 100 μg/chicken of the indicated recombinant plasmid or vacant pVAX-S-Flag was given by intramuscular injection and electroporation. After two-times immunization with two week intervals, the chickens were boosted with 5 × 10^7^ yeast cells expressing the indicated antigenic domain or HN or CD20 protein on day 7 post last immunization. On day 14 post boost, the immunized chickens were challenged with a total of 10^5^ EID_50_/0.1ml of NDV per chicken by intranasal inoculation as described previously [17]. The suvival of the challenged chickens was observed every day. Vaccination and challenge experiments were approved by the animal welfare committee of HVRI. Animals displaying severe clinical distress were sacrificed by exsanguination after knocking animals unconscious. The criteria for euthanasia were somnolence, apathy, akinesia or dyspnea as described previously [38]

### Statistical analysis

All data were analyzed with Origin version 8.0 software. Statistical analyses were performed using the paired one-tailed Student’s *t* test. *P* values less than 0.05 were considered statistically significant. Results were presented as mean values ± standard deviation (SD) of at least three repetitions.

## Results

### Construction and characterization of the combinatorial HN antigen yeast library from genotype VII NDV

**Fig 1:**
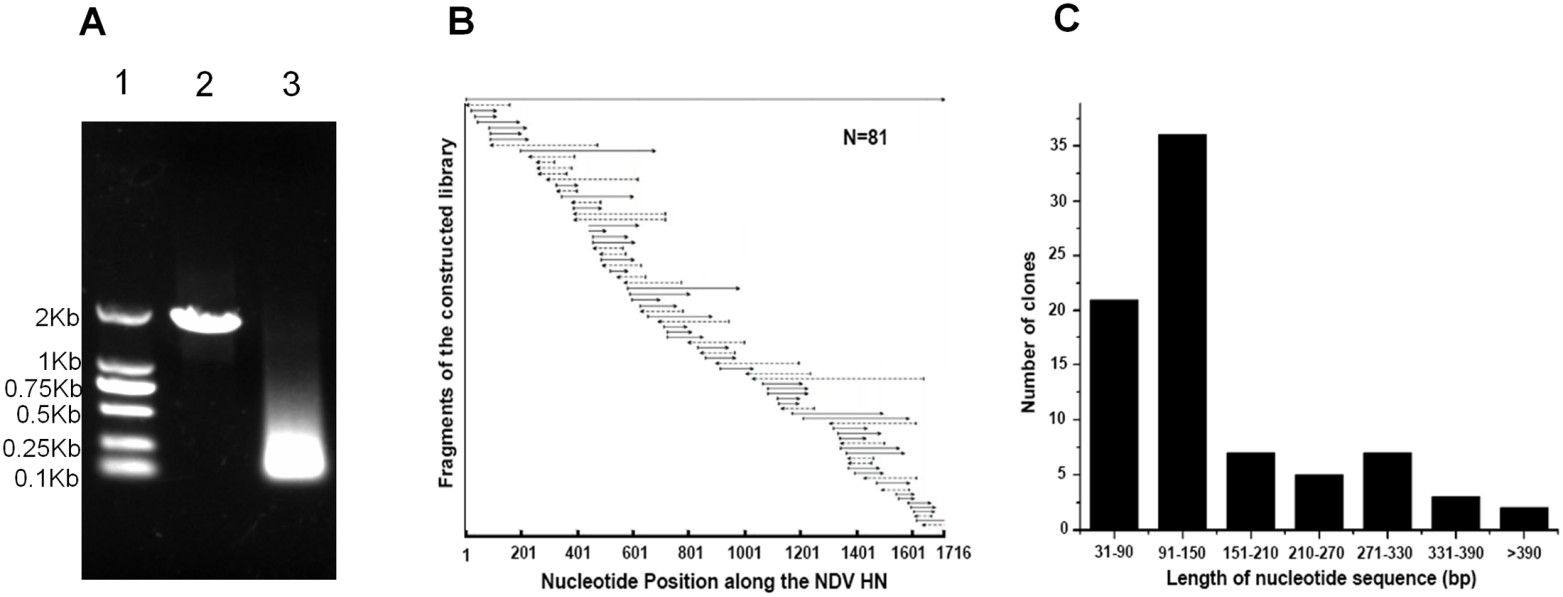
Construction and characterization of combinatorial HN antigen yeast library from genotype VII NDV. HN was amplified by PCR from genotype VII NDV and purified. The random HN fragments were produced by digestion of HN with DNase I and insertion into pCTCON-T vector to generate the combinatorial HN antigen yeast library. The quality of the constructed library was evaluated. (A) HN was amplified from genotype VII NDV and digested with DNase I. Lane 1, DL 2000 DNA ladder; Lane 2, HN PCR products; Lane 3, the randomly digested HN fragments. (B) 81 clones randomly selected from the yeast library were sequenced. The inserted HN fragments were aligned to the original HN nucleotide sequence. The solid and dashed lines refer to the HN fragments inserted into pCTCON-T vector in right and reverse direction, respectively. (C) Statistical analysis of the length of the inserted HN fragments in the sequenced clones from the yeast library.

To analyze the linear immunogenicity of the HN protein from genotype VII NDV, HN was amplified from the genome of strain JS2012 and randomly digested with DNase I to obtain 50 to 300 bp length fragments (Fig 1A). The obtained fragments were inserted into pCTCON-T and subsequently transformed into competent EBY-100 cells to generate a combinatorial HN antigen yeast library. The yeast library was titrated onto SDCAA Amp plates after a serial dilution. The resulting analysis showed over 2 × 10^5^ clones in the library. To evaluate the quality of the library, 81 clones randomly selected from the constructed library were sequenced. As shown in Fig 1B, the inserts covered and were randomly distributed along the full length of HN nucleotide sequence. The majority of the fragments in the library were between 52 and 210 bp in length (Fig 1C), of which, those sized 31–90 bp, 91–150 bp and 151–210 bp made up 25.9%, 44.4% and 8.6% of all sequenced fragments, respectively. These results indicated the successful construction of a yeast library suitable for the comprehensive analysis of linear antigenic regions on HN.

### Antigen screening from the combinatorial HN antigen yeast library using chicken immunosera against genotype VII NDV

**Fig 2:**
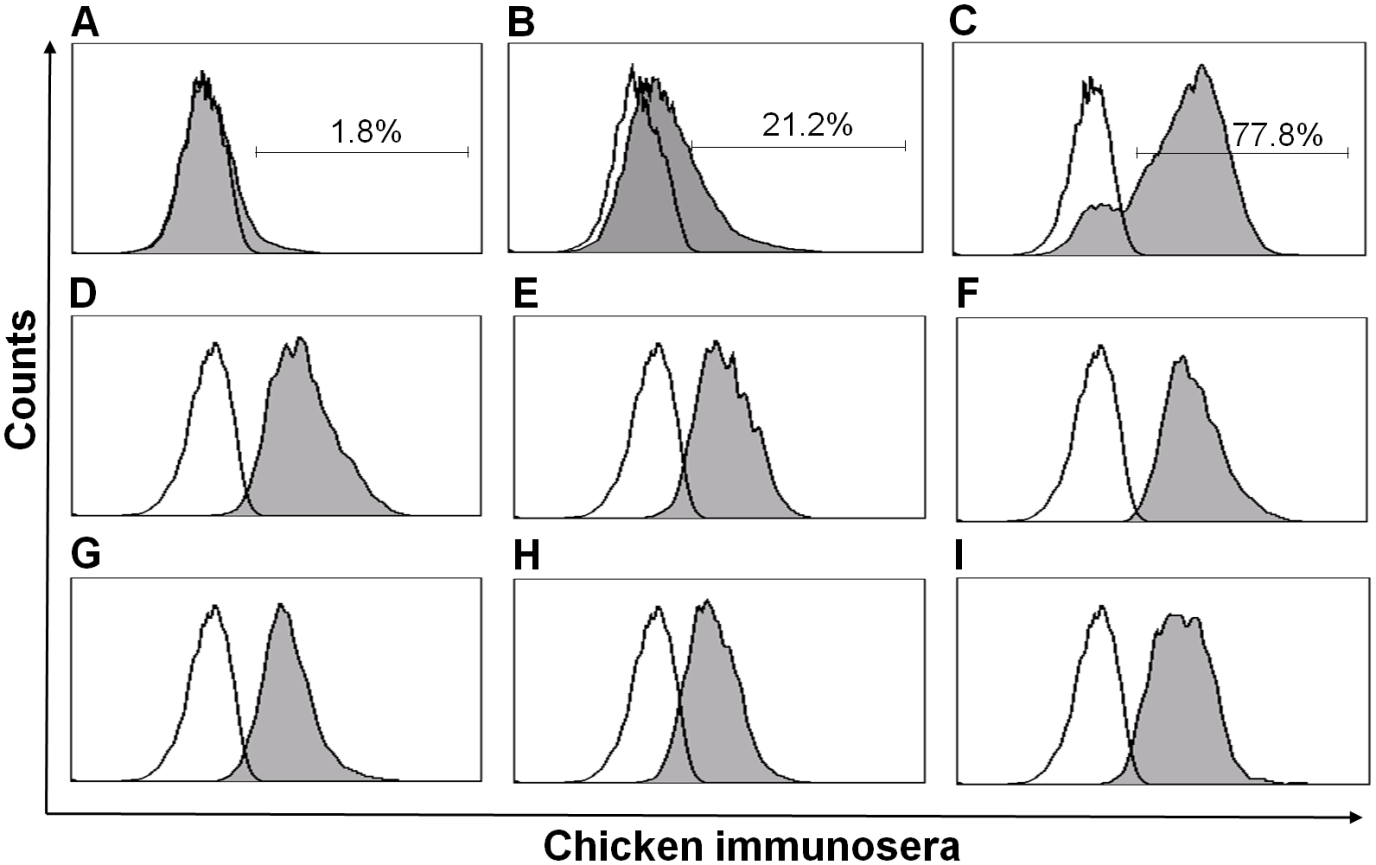
Sorting of the combinatorial HN antigen yeast library with FACS. The induced yeast cells were sorted and analyzed with FACS. (A) The induced combinatorial HN antigen yeast library was stained with the chicken immunosera against NDV and analyzed with FACS before the first sorting. (B) The first-sorted combinatorial HN antigen yeast library was analyzed with FACS after being stained with the chicken immunosera against NDV. (C) The second-sorted combinatorial HN antigen yeast library was analyzed with FACS after being stained with the chicken immunosera against NDV. (D—I) 6 clones randomly selected from the second-sorted combinatorial HN antigen yeast library were induced with the galactose overnight at 30°C. The expression of HN antigens was evaluated with FACS after being stained with the chicken immunosera against NDV. The open and gray histograms refer to the negative control and experimental groups, respectively.

To screen for the antigenic domains of HN, the combinatorial HN antigen yeast library was induced with 2% galactose for 36 h at 30°C, stained with chicken immunosera against genotype VII NDV and sorted by FACS twice. As shown in Fig 2, after a single round of sorting, the number of positive clones increased from 1.8% before sorting (Fig 2A) to 24.2% (Fig 2B). After a second round of sorting, the number of positive clones increased to 77.8% (Fig 2C). We validated these results by plating the selected clones on SDCAA Amp plates and randomly selecting six individual yeast clones for FACS analysis after inducing with galactose. As shown in Fig 2D-2I, each of the clones were positive. These results indicated the successful screening of HN antigen expressing yeast cells.

### Linear antigenic mapping of HN protein from genotype VII NDV

**Fig 3:**
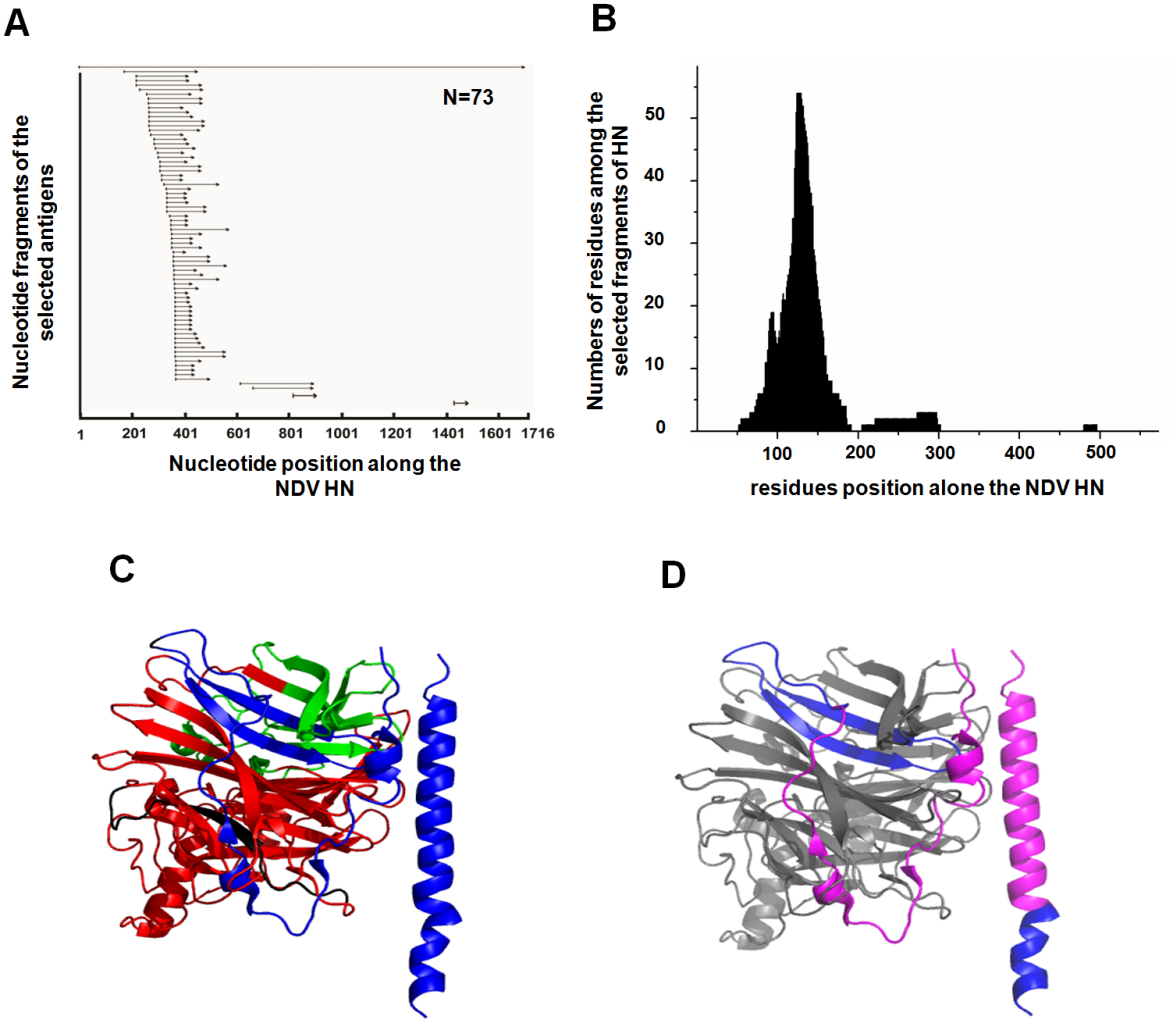
Analyses of linear antigenic regions on the HN protein from genotype VII NDV. 73 clones randomly selected from the sorted yeast library were sequenced. The nucleotide sequences of HN antigens were aligned to the full-length HN gene, and number of amino acid residues on the HN antigens was statistically analyzed along their corresponding position on the HN protein. (A) Nucleotide sequences of the sorted yeast clones overlapping and aligned to the original full-length HN sequence. (B) Number of amino acid residues among the selected fragments along their corresponding positions on the HN protein. (C) The sequenced HN antigens were docked on the crystal model of the extradomain of HN from NDV. The regions of 53–192 aa, 205–302 aa and 205–302 aa was marked with blue, green and black, respectively. (D) The P2 antigenic domain was docked on the crystal model. The amino residues marked with magenda and blue are appeared at high and low fraquencies in the sorted antigens located on P2 region, respectively.

To further determine how polyclonal antibody responses develop against the HN of genotype VII NDV in vivo, we analyzed the sorted antigenic fragments through sequencing. As shown in Fig 3A, 94.5% of the 73 randomly selected clones fell along the region containing amino acid residues 53–192. Three other clones fell in the region of amino residues 205–302 while one clone resided along the region of amino residues 480–496. Docking analysis of the sequenced HN antigens to the crystal model structure using the software PYMOL [39], as shown in Fig 3C, revealed the location of the amino residues on the stalk region and a part of globule head of HN protein [40]. Based on these results, we identified four distinct regions of the HN protein, P1 (1–52 aa), P2 (53–192 aa), P3 (193–302 aa) and P4 (303–571 aa). According to the statistical frequencies of the amino residues appearing on the sorted antigens, we further identified the P2 region as the dominant linear antigenic domain compared to the weak antigenic domains of the P1, P3 and P4 regions in chickens (Fig 3B). Based on the statistical analysis, the region 89–161 aa on the P2 antigenic domain appeared at high frequency among the sorted antigens compared to the regions 53–88 aa and 162–191 aa, which suggests that this region at 86–161 aa is perhaps the antigenic core of the P2 antigenic domain (Fig 3D).

### Validation of the mapped linear antigenic domains on the HN protein from genotype VII NDV

**Fig 4:**
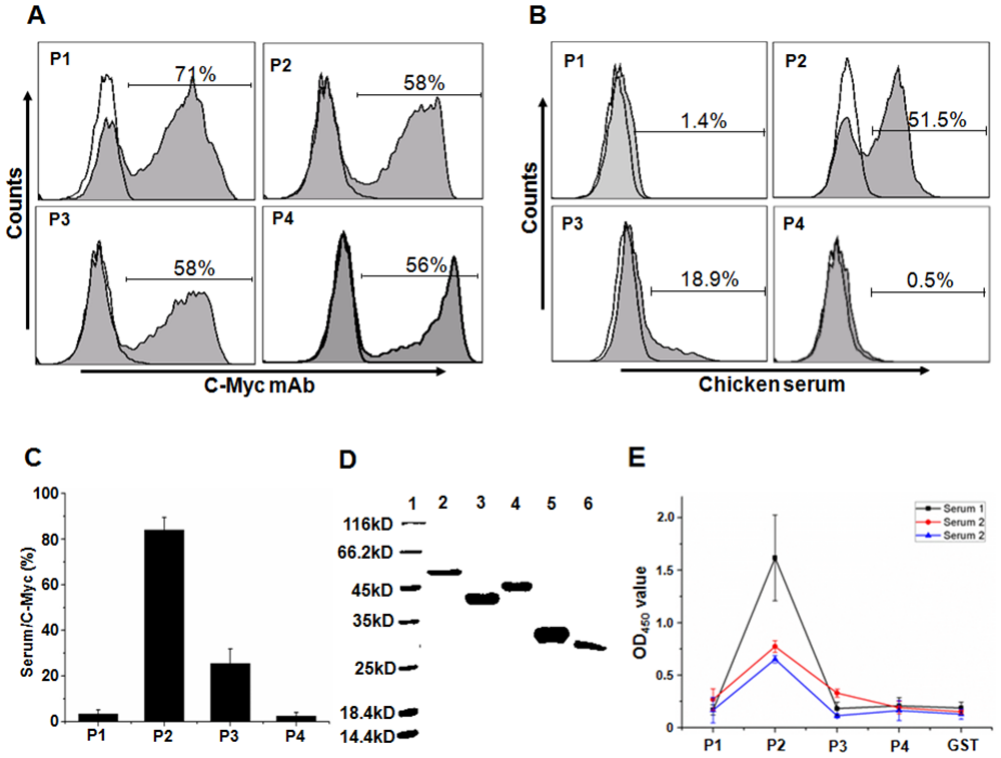
Validation of antigenic domains on the HN protein from genotype VII NDV. P1, P2, P3 and P4 were expressed on the yeast cells or in E. *Coli*. The antigenic domains of HN were validated with FACS and ELISA assays. (A) P1, P2, P3 and P4 expressed on the yeast cells were stained with antibodies against C-Myc tag and analyzed with FACS. (B) P1, P2, P3 and P4 expressed on the yeast cells were stained with chicken immunosera against the genotype VII NDV and analyzed with FACS. The figure shows a representative plot analyzed using one chicken immunoserum sample. (C) The positive rate of antigenic regions stained with chicken immunosera was normalized to those stained with antibodies against C-Myc tag (serum/C-Myc). The ration of serum/C-Myc for each antigenic region was plotted on the histogram and used as the indicator of robustness of responses. (D) P1, P2, P3 and P4 fused with GST were expressed in E. *Coli*. and purified. Their purity was confirmed with SDS-PAGE assay. Lane 1 is the protein marker, lane 2–6 were purified P4, P3, P2, P1 and GST, respectively. (E) The antigenic domain-specific antibodies in conralescent sera of chickens infected by the genotype VII NDV were evaluated with ELISA assay. GST served as the negative control.

To confirm the authenticity of the linear antigenic domains, we cloned P1 (excluding the transmembrane domain), P2, P3 and P4, into the pCTCON-2 vector and had them expressed on the yeast cell surface by induction with 2% galactose. Antibodies against the C-Myc tag fused to each fragment were used to confirm their expression. As shown in Fig 4A, FACS analysis revealed a distinct profile of positive C-Myc cells for each of the HN fragments, confirming their expression on the yeast cell surface. Yeast cells expressing P1, P2, P3 and P4 were then stained with chicken immunosera against NDV. As shown in Fig 4B, all defined linear antigenic domains reacted to the chicken immunosera with a varied robustness. Positive rates against the chicken immunosera were 1.4%, 51.5%, 18.9% and 0.5% for P1, P2, P3 and P4 regions, respectively. As it is a general phenomenon that some yeast cells will fail to express the antigenic domains during the induced culturing [26, 33, 34], the staining results by chicken immune sera will not directly indicate the robustness of the reaction. To adjust for this, we normalized the rate of positive staining with the chicken immunosera against NDV to the positive rate of antibody staining against C-Myc tag and used this to compare the robustness of the reaction among the mapped antigenic domains with the chicken immunosera. As shown in Fig 4C, the P2 region was the most robust antigenic domain compared to the other regions (P < 0.05). To further validate the mapped antigenic domains, P1, P2, P3 and P4 were expressed in E. *Coli*. and purified to establish an ELISA assay (Fig 4D and 4E). Three sera randomly selected from chickens infected by genotype VII NDV were evaluated using the ELISA assay. The result showed that P2 was highly reactive with all chicken immunosera against NDV (P < 0.05). Meanwhile, the reactions with P1, P3 and P4 to the three chicken immunosera were much less robust. This result was consistent with that of FACS analyses. Altogether, these results indicate that the P2 is the dominant linear antigenic domain on the HN protein.

### The dominant antigenic domain on the HN protein is sufficient to protect most chickens from genotype VII NDV infection

**Fig 5:**
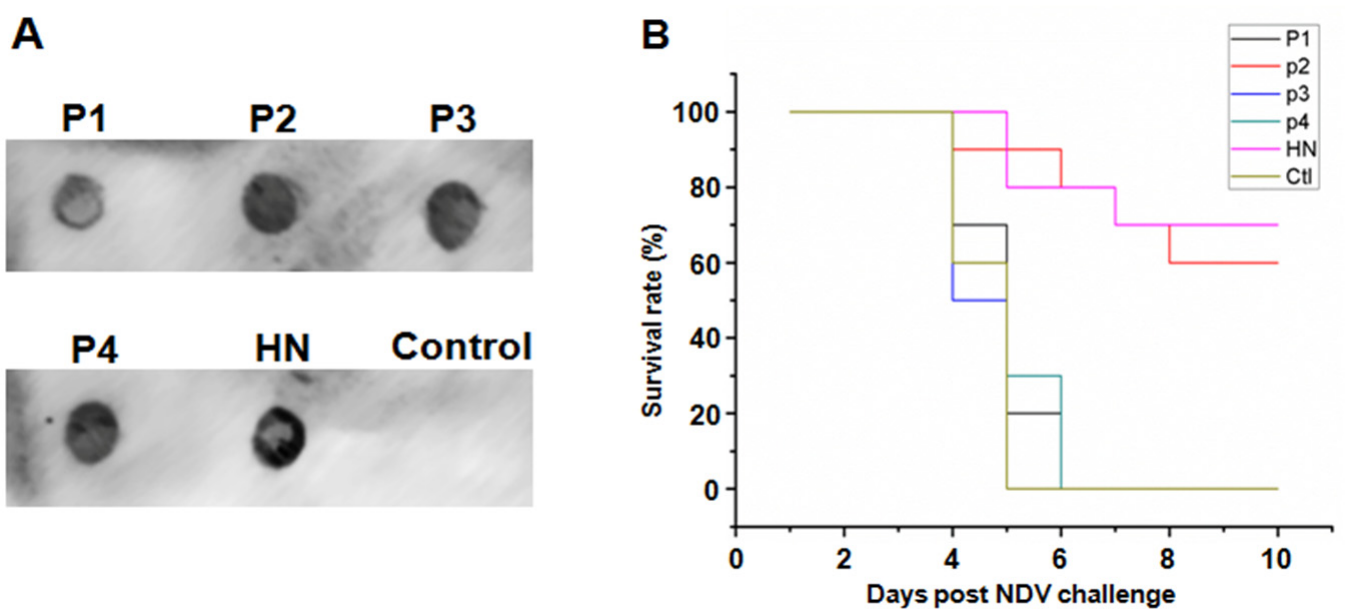
Evaluation of the protection from challenge with NDV in chickens with the antigenic domains. Recombinant plasmids pVAX-P1, pVAX-P2, pVAX-P3, pVAX-P4 and pVAX-HN, as well as pVAX-S-Flag serving as a control, were inoculated into SPF chickens by electroporation. The immunized chickens were challenged with lethal dose of NDV through intranasal inoculation after being boosted with the indicated antigenic protein-expressed yeast cells. (A) The recombinant plasmids were transfected into 293T cells. The antigenic proteins in the culture medium were precipitated by acetone and detected with dot-blot assays using anti-Flag tag monoclonal antibodies. Culture medium served as the control. (B) The survival of chickens after NDV challenge. “Ctl” refers to the control group.

To determine whether the antigenic domains protect chickens from NDV infection, individual antigenic domains and HN deleted the signal sequence were inserted into the eukaryotic expression vector pVAX-S-Flag to generate pVAX-P1, pVAX-P2, pVAX-P3, pVAX-P4 and pVAX-HN, respectively. After confirming their expression in mammalian cells with dot blot assay (Fig 5A), they were used to immunize chickens twice by electroporation. The immunized chickens were then given a boost of individual proteins expressed on the surface of yeast cells and challenged with 10^5^ EID_50_/chicken NDV viruses. As shown in Fig 5B, all chickens in P1, P3 and P4 groups, although they successfully generated specific antibodies (data not shown), as well as the control group, died by ten days post NDV challenge. In contrast, 60% (6/10) of the chickens immunized with P2 and 70% (7/10) of the chickens immunized with HN survived the NDV challenge, respectively. These results indicate that P2 region is not only the dominant antigenic domain, it is also the protective antigen on the HN protein which is able to elicit significant immune responses in vivo to protect the majority of chickens from NDV infection.

## Discussion

NDV is the causative agent of a highly contagious disease in poultry. There are several genotypes of NDV, of which genotype VII, velogenic strain, is the predominant type in circulation around China. In this study, we analyzed the linear antigenic domains on the HN protein from genotype VII NDV using a yeast surface display system. Our results revealed four distinct antigenic regions of HN, namely, P1 (2–52 aa), P2 (53-192aa), P3 (193-303aa) and P4 (304-571aa), respectively. We found P2 to be strongly reactive with chicken immunosera against genotype VII NDV and confirmed it to be the dominant linear antigenic domain of HN. Furthermore, immunization with the P2 antigen effectively protected the majority of chickens from challenge with genotype VII NDV. The antigenicity of the other regions were found to be much weaker and were unable to elicit protective antibody responses against NDV in chickens.

The yeast surface display system is generally used for the directed evolution of proteins [31, 41]. It was first reported in 2011 for comprehensive analyses of antibody responses against pathogens in vivo [33]. The quality of the constructed library, especially the length of the inserted fragments and the size of the library, is a determining factor for the successful analysis of antigenic domains. Previous reports have shown that 100–500 bp and 500–800 bp fragments are suitable for the analysis of linear and conformational antigenic domains, respectively, in yeast surface display systems [33, 34]. Although it was considered that the conformational structure of a native protein can be retained on the yeast cell surface using fragments 500–800 bp in length, we hypothesized that the substantial conformational epitopes will theoretically be destroyed when the length of the inserted fragment is shorter than the native protein. Therefore, the analyzing results of the conformational antigenic domains using the yeast surface display system may not reveal the true native characterization of the conformational antigenic domains. In contrast to this, linear epitopes will not be destroyed if the length of displayed fragments is longer than the length of epitopes which are generally less than 11 amino residues long. Thus, accurate analysis of linear antigenic domains is possible by controlling the length of inserted fragments. Considering these reasons, only the linear antigenic domains of the HN protein were mapped using the yeast surface display system. Theoretically, the ideal method for analysis of the linear antigenic domains would be to control the length of the inserts to about 33 bp. However, this would be difficult as the activity of DNase I cannot be controlled when randomly digesting DNA. In our library, almost all of the inserted fragments were within 300 bp in length, with over 70% of the fragments less than 150 bp (Fig 1C). This differed from previous yeast library constructions used for antigenic analyses [33]. Our use of shorter fragments has the obvious advantage of more accurate mapping of the linear antigenic domain (Fig 3). The library size is an important factor for the successful analysis of antigenic domains. If the library is too small, the analysis will not cover some possible peptides of a given antigen. A previous report showed that the size of a library containing fragments in a given length can be calculated based on the following formula: [(N-S)(N-S+1)-(N-L-1)(N-L)]/2 (N denotes nucleotide bases of a given antigen, while S and L denote nucleotide bases of small and large fragments inserted into the library) [42]. However, in our experience and Dr. Teng Zuo at Tsinghua University, the size of a library required for the entire antigenic analysis of a given antigen is actually smaller than the one calculated by the above formula [33]. Our practice indicates that the size of a library with 10 times the number of theoretical unique DNA fragments could cover all possible peptides and is enough for an entire antigenic analysis of a given antigen. Thus, although 2 × 10^5^ clones in our library is smaller than the size calculated based on the above formula (about 3 × 10^5^ clones), it is sufficient to cover all possible peptides between 20–100 aa because it is about 20 times the theoretical size of the library required for the antigenic analysis.

HN is one of the membrane proteins of NDV and responsible for the recognition and absorption of the virus to sialic acid-containing receptors on the target cells. Humoral immune responses against the HN protein are able to protect chickens from NDV infection, while reports have indicated that HN would be a good target for vaccine development [43–45]. Previous research looking to characterize the antigenic region on the HN protein of NDV have used monoclonal antibodies derived from mice [46, 47]. Considering the distant genetic diversity between mice and chickens, however, the defined antigenic regions using these mAbs may not be true in chickens. In contrast, the linear antigenic domains of HN defined here were identified using chicken immunosera against genotype VII NDV. We believe these results more likely reflect the authentic linear immunogenicity of HN in chickens. In our findings, the P2 region was able to response robustly to all chicken immunosera against NDV, compared to P1, P3 and P4. This allowed us to ascertain that P2 is the dominant antigenic domain on the HN protein. It also revealed that the immunogenicity between the different regions of HN varies greatly in chickens, a similar phenomenon that has previously been seen with the influenza virus [33, 34]. Based on these results, we believe that any future design of a vaccine against genotype VII NDV using the HN protein can omit specific regions such as P1, P3 and P4, due to their weak antigenicity in chickens (Fig 4).

The yeast library constructed here was composed of short HN fragments to screen for the linear epitopes of HN. Thus, the antigenic domains defined here were linear on the HN protein. While we found P2 to be the strongest linear antigenic domain on the HN protein, we think that the immunogenicity of P2 doesn’t correlated with its length, as the P4 region was longer despite analyses showing it to be much weaker. Our analysis focused on the linear antigens of HN, however, we cannot rule out the possible existence of conformational epitopes on the P2, P3 and P4 regions. In the immunization analysis, the P2 antigenic domain was able to protect the majority of chickens from NDV challenge (Fig 5B). The protective function of P2 may be explained by the location of P2 on the HN protein. Crystal structure analysis of HN from NDV reveals the ectodomain as a composition of the bundle stalk and globular head [39]. The bundle stalk domain of HN protein carries specificity determinants for F-protein activation, affects neuraminidase activity and contributes significantly to the oligomerization of the protein [48–50]. P2 contains the full bundle stalk spanning the residues 79–115 aa of HN [39]. In addition, a part of both the neuraminidase domain and the second receptor-binding site are located within the P2 region [39]. Antibodies against P2 will therefore block important functions of HN in the host [39] and explains the protective effect against NDV seen in our analyses. However, our results also showed that a substantial number of chickens (4/10) could not be protected by immunization with P2 (Fig 5B). Future analyses will be necessarily to explore this further.

In summary, this study successfully analyzed the comprehensive HN-specific antibody response against the linear epitopes in vivo in chickens using a yeast surface display system. The results indicated the P2 region to be the dominant antigenic domain on the HN protein and able to protect chickens from NDV infection. These results will provide important information for the understanding of humoral immune responses elicited by HN of NDV in vivo.
